# Pulmonary Toxicities Associated With the Use of Immune Checkpoint Inhibitors: An Update From the Immuno-Oncology Subgroup of the Neutropenia, Infection & Myelosuppression Study Group of the Multinational Association for Supportive Care in Cancer

**DOI:** 10.3389/fphar.2021.743582

**Published:** 2021-10-05

**Authors:** Bernardo L. Rapoport, Vickie R. Shannon, Tim Cooksley, Douglas B. Johnson, Lindsay Anderson, Ada G. Blidner, Gregory R. Tintinger, Ronald Anderson

**Affiliations:** ^1^ Department of Immunology, Faculty of Health Sciences, University of Pretoria, Pretoria, South Africa; ^2^ The Medical Oncology Centre of Rosebank, Johannesburg, South Africa; ^3^ The Multinational Association for Supportive Care in Cancer (MASCC), Immuno-Oncology Subgroup of the Neutropenia, Infection and Myelosuppression Study Group, Manchester, United Kingdom; ^4^ Department of Pulmonary Medicine, The University of Texas MD Anderson Cancer Center, Houston, TX, United States; ^5^ Manchester University Foundation Trust, Manchester, United Kingdom; ^6^ The Christie, University of Manchester, Manchester, United Kingdom; ^7^ Department of Medicine, Vanderbilt University Medical Centre and Vanderbilt Ingram Cancer Center, Nashville, TN, United States; ^8^ Department of Radiation Oncology, Steve Biko Academic Hospital, Faculty of Health Sciences, University of Pretoria, Pretoria, South Africa; ^9^ Laboratory of Immunopathology, Institute of Biology and Experimental Medicine, CONICET, Buenos Aires, Argentina; ^10^ Department of Internal Medicine, Steve Biko Academic Hospital, Faculty of Health Sciences, University of Pretoria, Pretoria, South Africa

**Keywords:** pneumonitis, immune checkpoint inhibitors, immune related adverse effects, anti-CTLA-4 antibodies, anti-PDL-1 monoclonal antibodies

## Abstract

The development of immune checkpoint inhibitors (ICIs) has revolutionized cancer treatment, with agents such as nivolumab, pembrolizumab, and cemiplimab targeting programmed cell death protein-1 (PD-1) and durvalumab, avelumab, and atezolizumab targeting PD-ligand 1 (PD-L1). Ipilimumab targets cytotoxic T lymphocyte-associated antigen-4 (CTLA-4). These inhibitors have shown remarkable efficacy in melanoma, lung cancer, urothelial cancer, and a variety of solid tumors, either as single agents or in combination with other anticancer modalities. Additional indications are continuing to evolve. Checkpoint inhibitors are associated with less toxicity when compared to chemotherapy. These agents enhance the antitumor immune response and produce side- effects known as immune-related adverse events (irAEs). Although the incidence of immune checkpoint inhibitor pneumonitis (ICI-Pneumonitis) is relatively low, this complication is likely to cause the delay or cessation of immunotherapy and, in severe cases, may be associated with treatment-related mortality. The primary mechanism of ICI-Pneumonitis remains unclear, but it is believed to be associated with the immune dysregulation caused by ICIs. The development of irAEs may be related to increased T cell activity against cross-antigens expressed in tumor and normal tissues. Treatment with ICIs is associated with an increased number of activated alveolar T cells and reduced activity of the anti-inflammatory Treg phenotype, leading to dysregulation of T cell activity. This review discusses the pathogenesis of alveolar pneumonitis and the incidence, diagnosis, and clinical management of pulmonary toxicity, as well as the pulmonary complications of ICIs, either as monotherapy or in combination with other anticancer modalities, such as thoracic radiotherapy.

## Introduction

Since FDA approval of the first ICI, ipilimumab, in 2011, a growing portfolio of monoclonal antibodies targeting the CTLA-4 and PD-1/PD-L1 pathways has emerged, which purport therapeutic advantage over a broad spectrum of cancers (Table 1). ICI-based therapies targeting CTLA-4 or PD-1/PD-L1 have led to significant improvements in overall survival and progression-free survival in patients with hematologic malignancies, as well as solid cancers, including Hodgkin’s lymphoma, melanoma, colon, renal cell, and Merkel cell carcinomas and non-small cell lung carcinoma (NSCLC) (Hodi et al., 2010; Brahmer et al., 2012; Ansell et al., 2015; Borghaei et al., 2015; Le et al., 2015; Motzer et al., 2015).

**TABLE 1:** Immune checkpoint inhibitor drug class and associated FDA approved tumor targets.

Target	Drug	FDA-approved
CTLA-4 (CD152) (Cytotoxic T-lymphocyte-associated antigen-4)	Ipilimumab (Yervoy)	Unresectable metastatic melanoma
		Renal cell carcinoma (with nivolumab)
PD-1 (CD279) (Programmed cell death protein-1)	Pembrolizumab (Keytruda)	Unresectable metastatic melanoma
		NSCLC-Stage IV
		Merkel cell carcinoma
		Hepatocellular carcinoma
		Gastric carcinoma
		GE junction carcinoma
		Cervical cancer
		Urothelial carcinoma
		Hodgkin lymphoma
	Nivolumab (Opdivo)	Unresectable metastatic melanoma
		Non-small cell lung cancer-Stage IV
		Small cell lung cancer (extensive disease)
		Hepatocellular carcinoma
		Renal cell carcinoma (with ipilimumab)
	Cemiplimab	Advanced squamous cell carcinoma-skin
PD-L1 (CD274, B7 homolog) (Programmed cell death –ligand 1)	Atezolizumab	Non-small cell lung cancer-Stage IV
		Small cell lung cancer (extensive disease)
		Breast carcinoma (triple negative)
	Avelumab	Merkel cell carcinoma
	Durvalumab	Urothelial cancer

With the expansion of ICI-targeted therapies and the development of complex combination and multimodal therapies, there has been increasing recognition of a unique spectrum of associated end-organ toxicities, referred to as immune-related adverse events (irAEs). Although the precise pathophysiologic mechanism of lung injury associated with ICIs remains under rigorous investigation, it is thought that these therapies act as a double-edged sword in which release of regulatory controls is responsible for both the therapeutic efficacy of ICI therapy and the driver of irAEs. The incidence of severe or life-threatening irAEs (grade ≥ 3) ranges from 20 to 30% for patients receiving anti-CTLA-4 and 10–15% for patients treated with anti-PD-1/anti-PD-L1 agents. The incidence of these severe irAEs is highest (55%) for patients treated with the combination of anti-CTLA-4/PD-1 or anti-CTLA-4/PD-L1. These irAEs may affect virtually every organ system (Darnell et al., 2020). Lung irAEs were rarely described in early clinical trials, but have become increasingly recognized as clinically significant, becoming more complex as ICI-containing regimens have expanded (Topalian et al., 2012). Pneumonitis is the primary manifestation of lung irAEs. Pleural effusions, airway disease, and mediastinal lymphadenopathy associated with sarcoid-like reactions have also been described (Nishino et al., 2017a; Suresh et al., 2018b; Gkiozos et al., 2018; Rambhia et al., 2019; Mitropoulou et al., 2020; Naidoo et al., 2020; Nobashi et al., 2020; Shannon, 2020; Zhu et al., 2020). Most cases of ICI-related lung injury are mild, but these events may be irreversible and fatal. In fact, lung-related irAEs are the most common cause of ICI interruption and ICI-related mortality (Nishino et al., 2016a; Wang et al., 2018). Thus, knowledge regarding key characteristics and optimal treatment of lung irAEs is critical to good outcomes. This review provides an updated overview of the biology of ICIs, the epidemiology, mechanisms, clinicopathologic features, diagnosis, and management of lung irAEs.

## Biology of Immune Checkpoints and Rationale in Cancer Therapeutics

Compelling evidence gathered from studies highlighting key roles of the immune system in cancer development has generated enormous interest in the complex network of regulatory pathways that govern immune homeostasis. Uncontrolled or excessive immune responses to foreign pathogens or overexpressed self-antigens may promote inflammatory tissue damage and autoimmunity. Thus, immune homeostasis, which is tightly regulated by a balance between co-stimulatory and inhibitory signals collectively known as immune checkpoints, is critical for host survival. Cytotoxic T-lymphocyte antigen-4 (CTLA-4) and its corresponding ligand B7.1/B7.2 and PD-1 and its major ligands, PD-L1 and PD-L2, are dominant immune checkpoint pathways that are essential modulators of immune activation. These inhibitory checkpoint pathways play critical roles in maintaining immune tolerance and T-cell responses within a desired physiologic range, thereby ensuring more uniform and controlled immune reactions and avoiding immune hyper-responsiveness and autoimmunity (Waterhouse et al., 1995).

Engagement of CTLA-4 with its cognate ligands is induced early, shortly after T cell activation (during T-cell priming), and effectively down-regulates immune T-cell functions (Brunner et al., 1999). CTLA-4 expression occurs primarily on activated T-cells within lymphoid tissues during T-cell priming, but may also attenuate T-cell activation within peripheral tissues (Ritprajak and Azuma, 2015). The binding ligands for PD-1 are widely expressed. PD-L1 expression is abundant in immune cells, such as activated T-cells, B cells, dendritic cells, macrophages, myeloid cells, and NK cells (Ritprajak and Azuma, 2015). Normal parenchymal tissues, including the heart, skeletal muscle, placenta, and lung tissues, express PD-L1 at high levels. In addition, induction of PD-L1 protein in various non-lymphoid tissue cells, including epithelial, endothelial, and smooth muscle cells, occurs in response to inflammatory cytokines released by damaged tissues (Freeman et al., 2000; Liang et al., 2003; Rodig et al., 2003; Youngnak-Piboonratanakit et al., 2006). PD-L2 expression is limited to activated dendritic cells and macrophages. PD-1/PD-L1 and PD-1/PD-L2 interactions inhibit effector T-cell function and are dominant in the peripheral tissues and tumor microenvironments (Parry et al., 2005; Keir et al., 2006; Weber, 2010; Wei et al., 2018). The differential contributions of the CTLA-4 and PD-1 co-inhibitory receptors create diverse expression phenotypes, which may explain the varied efficacy of ICIs across tumor types, as well as their distinct toxicity profiles.

Cancer cells often co-opt immune suppressive and tolerance mechanisms as a strategy to escape immune destruction. For example, by activating CTLA-4, PD-1, and PD-L1 inhibitory signals, cancer cells can subvert or disarm immune regulatory pathways, leading to increased tumor survival (Ribas and Wolchok, 2018; Tang et al., 2018). In addition to overexpression of inhibitory checkpoint proteins, tumor cells also activate intrinsic cellular signals that enhance cancer cell survival. Immune checkpoint inhibitors effectively block these proteins, thereby restoring T-cell function and augmenting immune attack and tumor killing (Butte et al., 2007; Escors et al., 2018; Ribas and Wolchok, 2018). The therapeutic potential of ICIs for treating numerous cancers is well recognized, but these agents may also induce significant irAEs, with the lung being an important target.

### Incidence of Immune Checkpoint Inhibitor-Mediated Pneumonitis

The incidence of pneumonitis in patients treated with ICIs varies in different analyses. One large meta-analysis involving 16 trials and 6,208 patients reported an incidence of 4% for any pulmonary toxicity and 1.5% for high-grade pneumonitis, which appeared similar across PD-1 and PD-L1 inhibitors (Sun et al., 2019). A subsequent, larger meta-analysis showed that anti-PD-1/PD-L1 was associated with a 5-6 fold higher risk of pneumonitis compared with cytotoxic chemotherapy (Huang et al., 2019). Notably, the odds ratio for pneumonitis for the combination of ipilimumab/nivolumab was approximately twice as high as single-agent anti-PD-1 therapy. Real-world studies have suggested similar incidences in NSCLC and melanoma patients, at approximately 5% for anti-PD-1/PD-L1 monotherapy and 10% for combination ipilimumab and nivolumab (Naidoo et al., 2017). Although fatal toxicities are uncommon (approximately 0.4% for single-agent anti-PD-1/PD-L1 and 1.2% for combination ipilimumab and nivolumab), pneumonitis likely comprises the most frequent fatal event, accounting for 35% of fatal toxicities from anti-PD-1/PD-L1 (Wang et al., 2018).

Interestingly, combinations involving anti-PD-1/PD-L1 with either chemotherapy or vascular endothelial growth factor (VEGF) inhibitors may actually have a slightly lower incidence of pneumonitis, although these regimens have generally not been directly compared with anti-PD-1 monotherapy. The meta-analysis described above suggested that the combination of pembrolizumab and chemotherapy had a lower risk of pneumonitis compared with anti-PD-1 therapy alone (although higher than chemotherapy) (Huang et al., 2019). Randomized studies, however, have shown that pneumonitis occurs in 5–8% of patients treated with pembrolizumab and chemotherapy, which appears broadly similar to that observed with single-agent therapy (Gadgeel et al., 2020; Paz-Ares et al., 2020). Pneumonitis appears perhaps slightly less common for the combination of pembrolizumab and axitinib (3%, with one fatal case) compared with single-agent anti-PD-1/PD-L1, although these have not been directly compared prospectively (Atkins et al., 2021).

### Mechanisms of Alveolar Damage in Pneumonitis (Non-Immune Checkpoint Inhibitor-mediated)

To gain insight into the mechanisms of alveolar damage in ICI-pneumonitis, we will briefly discuss the mechanisms of non-ICI associated types of pneumonitis. Pneumonitis is a condition that encompasses a diverse spectrum of acute and chronic inflammatory etiologies that evoke immune-mediated damage to alveoli with resultant respiratory dysfunction. Triggers of pneumonitis include a variety of airborne organic and inorganic irritants, as well as radiation therapy, aspiration and medications delivered by any route, resulting in non-IgE-mediated hypersensitivity pneumonitis (HP, also known as extrinsic allergic alveolitis), radiation-induced lung injury (RILI), and drug-induced interstitial lung disease (DILD), respectively. Pneumonitis may vary with respect to severity and duration (sub-acute, acute, and chronic) and the more severe forms of these pulmonary disorders may lead to development of extensive fibrosis as a complication of disease progression. Acute interstitial pneumonitis (AIP, Hamman-Rich syndrome), on the other hand, is a distinct entity of unknown etiology that is rapidly progressive. It is an uncommon condition that clinically resembles the acute respiratory distress syndrome (ARDS) and is associated with a high mortality of up to 60% (Mastan et al., 2018).

Although not entirely clear, the immunopathogenesis of HP appears to involve Th17 cells that are activated following exposure to the offending irritant/antigen in the lungs. The resultant production of IL-17A promotes IL-8-mediated influx of neutrophils into the lungs with resultant inflammation-mediated alveolar damage (Pardo et al., 2000; Girard et al., 2011; Hasan et al., 2013; Costabel et al., 2020; Elmore et al., 2020). Neutrophil recruitment to the lungs may be exacerbated by low numbers of pulmonary regulatory T cells (Tregs) (Girard et al., 2011; Elmore et al., 2020) and by the production of IgG antibodies to the offending stimulus (Costabel et al., 2020).

Some patients with HP develop chronic, severe disease that is associated with the occurrence of fibrosis. Although the underlying immunopathogenesis remains to be established, an association with co-existent autoimmune disease has been described. In this context, one study has reported a high prevalence of autoimmune thyroiditis in patients with chronic HP (25.6% in patients vs. 10.7% in matched control subjects, *p* < 0.0001), which was found to be a significant predictor of mortality (HR = 3.39, *p* = 0,012) (Adegunsoye et al., 2017). Similar findings have been reported in a subset of HP patients who expressed the DRB1*03:01-DQB1*02:01 HLA haplotype that was associated with a significant risk of development of antinuclear antibodies (OR = 19.23; *p* = 0.0088), while expression of the HLA-DRB1*03:01 allele was associated with higher mortality (OR = 5.9; *p* = 0.043) (Buendía-Roldán et al., 2020). Two other recent studies have described associations between seropositivity for antinuclear antibodies in patients with HP that was correlated with disease severity and progression (Adegunsoye et al., 2015; Bonella et al., 2019).

Giuranno et al. (2019) have recently described in detail events involved in the pathogenesis of RILI. Among others, the most prominent mechanisms of pulmonary damage involve radiation-associated generation of reactive oxygen species (ROS), which are cytotoxic for both type I and type II pneumocytes (Giuranno et al., 2019). Type I cells line the alveoli and maintain structural integrity, while type II pneumocytes produce the protective, lower airway lining lipoproteins, surfactants A and D. In addition, radiation-induced damage to resident cells in the airways also results in the release of damage-associated molecular patterns (DAMPs). These “alarmins” initiate the recruitment of cells of the innate immune system, particularly neutrophils, which initiate alveolar damage via the release of indiscriminate ROS and proteinases, as well as neutrophil extracellular traps (NETs) (Giuranno et al., 2019). In this context, the alarmin, high-mobility group box 1 (HMGB1) and the chemokine, IL-8, are major inducers of NETosis (Tadie et al., 2013; An et al., 2019) with NETs having been described as playing a key role in the “development of ILD of different etiologies” (Porto and Stein, 2016).

Drug-induced interstitial lung disease may manifest as an acute interstitial pneumonitis mediated by cytotoxic and immunological mechanisms (Matsuno, 2012). Reactive oxidants that are toxic to the lung and cytokine-mediated activation of inflammatory cells play important roles in promoting DILD.

### Putative Mechanisms of Immune Checkpoint Blockade-mediated Pneumonitis

Although several clinical risk factors for the development of ICI-Pneumonitis have been identified, little is known about the immunopathogenesis of this condition. Known risk factors include type of malignancy (not surprisingly NSCLC, as well as renal cell carcinoma), bronchial asthma, chronic obstructive pulmonary disease (COPD), and prior radiotherapy (Giuranno et al., 2019). Although not rigorously explored, measurement of systemic biomarkers of sub-clinical inflammation such as the circulating neutrophil count, interleukin (IL)-6, and C-reactive protein (CRP) may help identify those at risk, while, as mentioned earlier, measurement of thyroid and antinuclear autoantibodies may also have predictive potential (Adegunsoye et al., 2015; Adegunsoye et al., 2017; Bonella et al., 2019; Buendía-Roldán et al., 2020).

The most probable mechanism of induction of ICI-Pneumonitis is the recovery of pulmonary T cell reactivity to inhaled antigens, autoantigens, or microbial products following ICI-mediated reversal of the constraints (CTLA-4 and PD-1) imposed on Th1 and Th17 cells by Tregs. In this context, a recent report by Wang et al. is noteworthy (Wang et al., 2020). These authors measured the levels of the counteracting cytokines IL-17A (pro-inflammatory) and IL-35 (immunosuppressive, produced by Tregs) in bronchoalveolar lavage fluid (BALF) from patients (*n* = 13) with NSCLC who were experiencing episodes of ICI-Pneumonitis. ICI-treated, non-ICI-Pneumonitis NSCLC patients (*n* = 20) were included as controls. The test cytokines and the percentages of Th1, Th2, Th17, and Tregs were also measured in blood. The authors noted higher frequencies of Th1 and Th17 cells in blood that correlated positively with IL-17A (blood and BALF) and higher ratios of Th17 cells to Tregs during development of ICI-Pneumonitis. Although the numbers of patients recruited to the study were small and given that independent confirmation of these findings is necessary, they nevertheless appear to implicate Th17 cells in the immunopathogenesis of ICI-Pneumonitis, as well as a counteracting role for IL-35 (Wang et al., 2020). Partially confirming these results, another report by Suresh et al. revealed that high lymphocytosis was associated with ICI-Pneumonitis in BALF from ICI-treated patients with lung and skin cancers (Suresh et al., 2019b). While healthy individuals and ICI-treated patients that did not develop ICI-Pneumonitis had 10% lymphocytes and 85% macrophages as predominant BALF immune populations, ICI-Pneumonitis in ICI-treated patients was associated with a lymphocytosis of greater than 20%. To investigate the phenotype of these lymphocytes, the authors performed a comprehensive flow cytometry analysis and found that central memory and TNF-α^high^IFN-γ^high^ CD8 T cells were enhanced in ICI-Pneumonitis patients, in the setting of attenuation of immunosuppressive CTLA4^high^PD1^high^ Treg cells.

Moreover, they also observed an increase in the numbers of IL-1β^hi^TNF-α^hi^CD-11b^hi^CD14^int^CD16^int^ inflammatory myeloid cells (Suresh et al., 2019b). Although this work may not have confirmed a clear role for IL-17A, it became evident that in response to ICI treatment, patients that develop ICI-Pneumonitis recruit increased numbers of inflammatory cells to the lungs, while immunoregulatory populations decreased in number and potency.

Additional mechanisms that may be involved in the immunopathogenesis of ICI-Pneumonitis include: 1) autoantibodies; 2) auto-inflammatory cytokine cascades; 3) neutralization of type M2 macrophages and myeloid-derived suppressor cells; and 4) transition of the pulmonary microbiome to a pro-inflammatory phenotype.

### Clinical Approach to Immune Checkpoint Inhibitor-Mediated Pneumonitis

The onset of ICI-Pneumonitis is most often heralded by symptoms of dyspnea (53–79%), dry cough (35–88%), low-grade fever (12%), and chest pain (7%). The time from the administration of the first dose of an ICI to the occurrence of ICI-Pneumonitis varies widely from 1.9 to 24 months, with a median time of 2.8 months (Hassel et al., 2017; Wang et al., 2021). Earlier occurrences have been reported with combination therapies (median 2.7 vs. 4.6 months) and among patients with lung cancer (2.1 months) compared to melanoma (5.1 months) (Zhu et al., 2020).

Pneumonitis terminology is standardized, and associated symptoms are graded on a 5-point scale in accordance with the common terminology criteria for adverse events (CTCAE). Compatible radiographic changes occurring in the absence of symptoms (grade 1) or in the presence of mild symptoms that do not limit normal activities of daily living (grade 2) are designated as mild-to-moderate (low-grade) pneumonitis. High-grade pneumonitis is indicated by severe or medically significant symptoms that are not immediately life-threatening (grade 3), while life-threatening symptoms that require urgent medical attention are considered grade 4 pneumonitis. Pneumonitis-related death is designated as grade 5 (Table 2) (Basch et al., 2014; Kluetz et al., 2016). Mild-to moderate (grade 1–2) pneumonitis accounts for approximately 73–85% of ICI-Pneumonitis across tumor types. Higher rates of severe and fatal pneumonitis have been reported among patients with NSCLC, pre-existing fibrotic lung disease, and among patients treated with dual ICI therapy, ICI plus potentially pneumotoxic chemotherapy combinations and following talc slurry pleurodesis (Suresh et al., 2018b; Sakata et al., 2018; Wang et al., 2018; Moey et al., 2020; Yamagata et al., 2021; Yu et al., 2021).

**TABLE 2:** National cancer institute common terminology criteria for adverse events (CTCAE) pneumonitis grading system.

CTCAE grade	Clinical symptoms	Associated radiologic findings
I	Asymptomatic	Infiltrates confined to one lobe of the lung or less than 25% of the lung parenchyma
II	New respiratory symptoms or aggravation of existing symptoms, including dyspnea, cough, chest pain, low-grade fever, or increased oxygen requirements	Infiltrates involve 25–50% of the lung parenchyma on chest computed tomography (CT)
III	Severe symptoms that limit activities of daily living	Infiltrates involve all lung lobes or >50% of the lung parenchyma
IV	Life threatening symptoms, severe hypoxia requiring urgent intervention (ie, tracheostomy, or intubation with ventilator support)	Infiltrates involve all lung lobes or >50% of the lung parenchyma
V	Death	—

**FIGURE 1:**
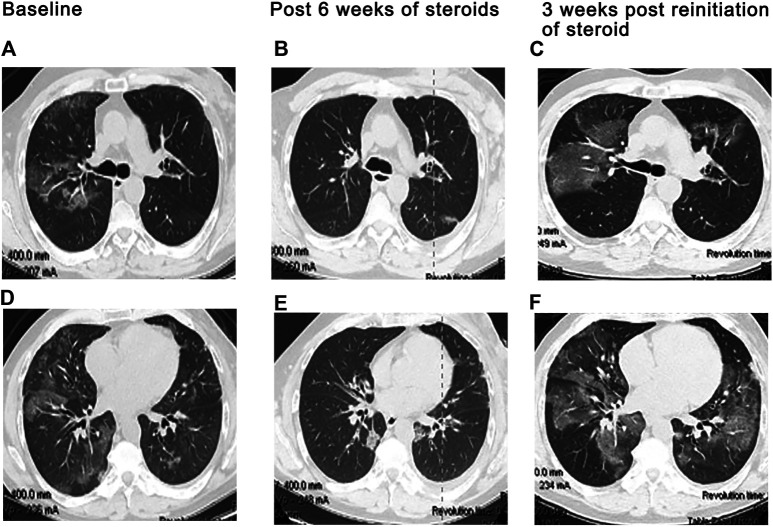
Exacerbation of preexisting interstitial lung disease. A 68 year-old man with a history of mild idiopathic pulmonary fibrosis and prostate cancer. Severe dyspnea and cough developed 19 days following cycle 1 of ipilimumab plus hormonal therapy, given per protocol. Chest CT at baseline **(A,C)** demonstrates subpleural ground glass and reticular opacities consistent IPF. CT imaging at presentation **(B,D)** shows interval development of poorly marginated scattered consolidative and ground glass opacities consistent with pneumonitis. In addition, there is bilateral diffuse thickening of the interlobular septa associated with architectural distortion suggesting progression of underlying fibrosis. Findings suggest that even mild preexisting lung disease may potentiate severe pneumonitis and worsening fibrotic disease following ICI therapy.

**FIGURE 2:**
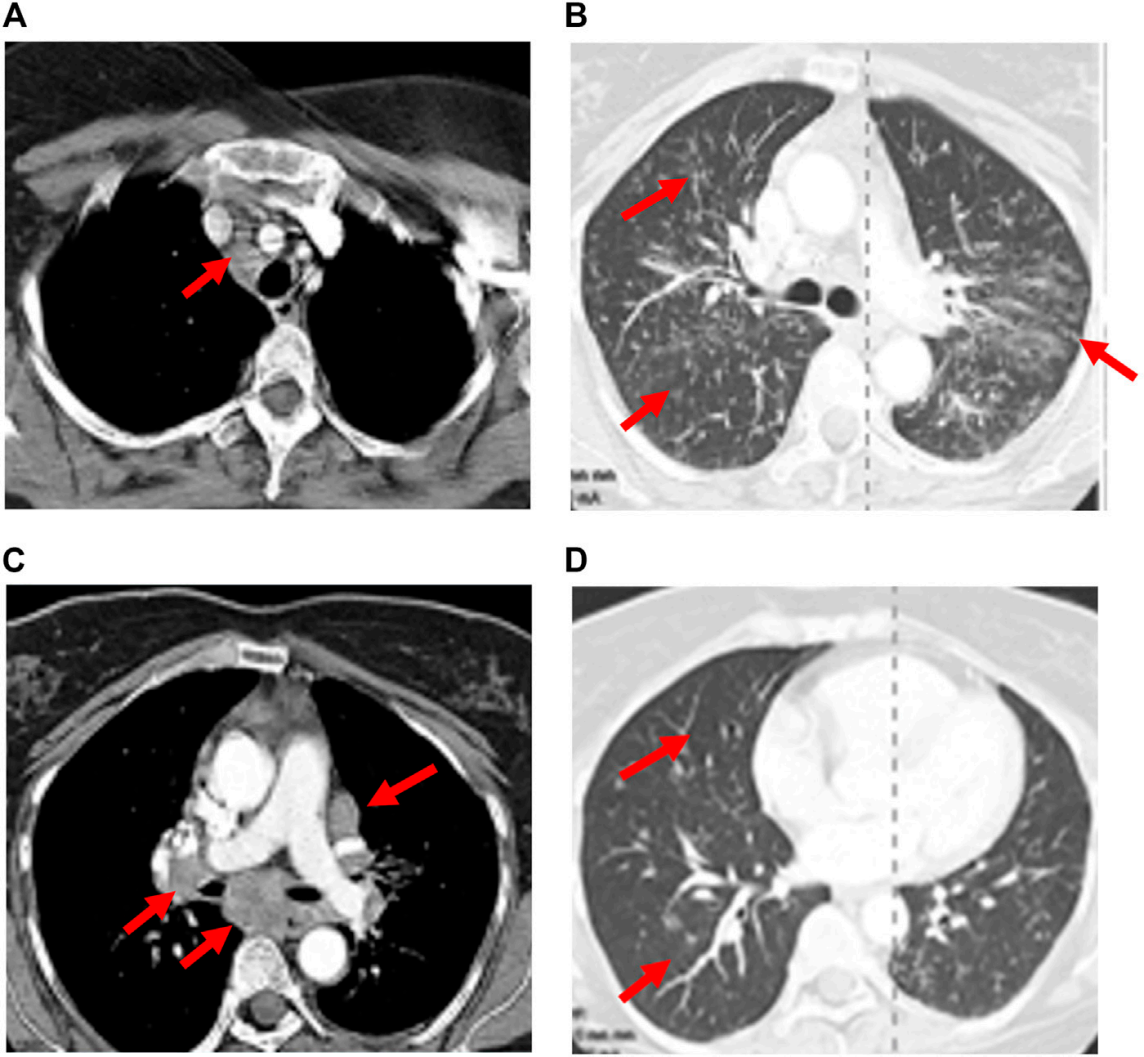
Recrudescence of pneumonitis with drug rechallenge. A 47-year-old man presented with dry cough and dyspnea 11 weeks after initiation of Nivolumab for treatment of non-Hodgkin lymphoma. CT scan showed patchy bilateral ground glass changes **(A,D)**. Bronchoscopy with biopsy confirmed organizing pneumonia associated with grade II pneumonitis and the patient was started on high dose steroids. Clinical and radiographic improvement was seen 6 weeks later after tapering off steroids **(B,E)**. Nivolumab was resumed. Three weeks later, the patient returned with increased respiratory symptoms and hypoxia. CT imaging demonstrated bilateral infiltrates which were worse than initial presentation **(C,F)**.

A diverse spectrum of radiographic features of pneumonitis may be found on CT imaging of the thorax. Five major subtypes have been recognized (Table 3): organizing pneumonia (OP), ground-glass opacities (GGO), nonspecific interstitial pneumonitis (NSIP) (Figure 1), hypersensitivity pneumonitis (HP), and acute interstitial pneumonia (AIP) (Figure 2) associated with the acute respiratory distress syndrome (ARDS) (Nishino et al., 2016b; Naidoo et al., 2017). The radiologic subtypes are consistent throughout the clinical course of ICI-Pneumonitis, although multiple histologic subtypes may coexist in the same patient, while evolution to other histologic subtypes over the course of ICI-Pneumonitis has been reported (Koelzer et al., 2016; Naidoo et al., 2017). Radiographic patterns of pneumonitis may correlate with clinical severity and grades of pneumonitis. AIP and ARDS correlate with more severe pneumonitis and a worse prognosis followed by OP, while NSIP and HP are associated with lower-grade pneumonitis. Thus, radiographic correlates of pneumonitis are significant prognosticators of pneumonitis outcomes and may assist in the management, follow-up, and monitoring of these patients (Nishino et al., 2016a; Nishino et al., 2017b).

**TABLE 3:** Pneumonitis patterns associated with ICI-related lung injury.

Histopathologic correlate	Clinical signs and symptoms	CT description	Usual CTCAE grade	CT findings
Organizing pneumonia (OP)	Nonproductive cough, dyspnea, weight loss, typically develop over<2 months	Patchy areas of consolidation or ground-glass opacities, localize most often to lung periphery; solitary opacities,multi-lobar alveolar opacities, or infiltrative opacities can be seen; reverse halo sign	2	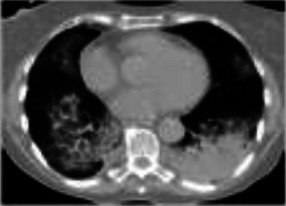
Nonspecific Interstitial Pneumonitis (NSIP)	Nonproductive cough, dyspnea typically develop over weeks to months. Bibasilar crackles	Bilateral, reticular markings, traction bronchiectasis, and ground-glass opacities typically localizes to the lower lung zones; subpleural sparing	1–2	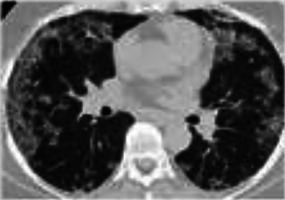
Ground Glass opacities	Nonproductive cough, dyspnea typically develop over weeks to months. Bibasilar crackles	Diffuse predominantly peripheral airspace opacities. May be predominantly peripheral or subpleural in location	1–2	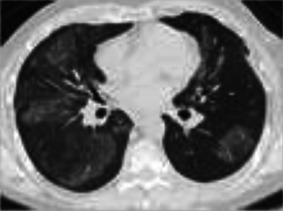
Hypersensitivity Pneumonitis (HP)	Dyspnea non-productive cough fatigue typically develop within weeks to months	Diffuse or centrilobular micronodular or reticular opacities are most prominent in mid-to-upper lobe predominance; air trapping	1–2	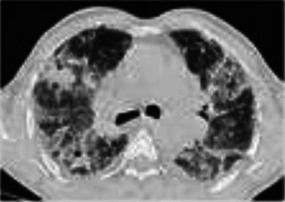
Diffuse Alveolar Damage (DAD)/Acute Respiratory Distress Syndrome	Acute onset of progressive dyspnea cough typically develops over days to weeks	Bilateral diffuse airspace opacities are more prominent in the dependent areas of the lungs	3–4	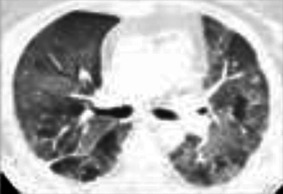

## Differential Diagnosis

The differential diagnosis of ICI-Pneumonitis poses clinical challenges, particularly in the setting of pre-existing lung disease, radiation pneumonitis, congestive heart failure, and lymphangitic spread of tumor. In addition, infectious pneumonia caused by bacteria, viruses, tuberculosis (van Eeden et al., 2019), and fungi may mimic ICI-Pneumonitis. Clinical and radiographic clues may help to distinguish ICI-Pneumonitis from infectious pneumonia. For example, fever, leukocytosis, and discolored sputum are clinical hallmarks of lung infection, but uncommon in ICI-Pneumonitis. ICI-Pneumonitis-related fevers, when they occur, are more often low grade. Imaging manifestations of infectious pneumonias, including multi-lobar consolidations and ground glass opacities, may be indistinguishable from ICI-Pneumonitis. Hemoptysis, weight loss, and increasing serum tumor markers along with new or increasing nodular shadows and interlobular septal thickening are helpful clinical and radiographic clues that favor cancer progression. The challenges in distinguishing ICI-Pneumonitis from radiation pneumonitis are particularly relevant, especially in the setting of ICI-Pneumonitis following concurrent chemoradiation therapy.

### Radiation Therapy, Immune Checkpoints, and Pneumonitis: Clinical Implications

Radiation therapy (RT) is a standard treatment modality for certain thoracic malignancies in both the curative and palliative settings. Radiation pneumonitis is an acute manifestation of radiation-induced lung damage and typically occurs 4–12 weeks after treatment is completed. Characteristic radiologic features include ground-glass opacities and airspace consolidation, usually located within or at the edge of the irradiated field. Dyspnea, cough and chest pain are typical symptoms, which can be challenging to distinguish from other comorbidities, such as COPD.

The theory that a synergistic therapeutic effect exists between prior chest RT and ICIs has previously been reported (Herrera et al., 2017). This has led to the implementation of studies that aim to establish the safety and efficacy of concurrent radio-immunotherapy. On the other hand, however, there are studies that have suggested that prior chest RT is a risk factor for the development of ICI-Pneumonitis due to underlying radiation-induced lung damage (Zhai et al., 2020). The exact mechanism underpinning this event remains an area of active interest.

The advent of ICIs has impacted significantly on the outcome of patients with unresectable stage III and metastatic NSCLC in particular. These patients require multimodality treatment and receive RT in either the curative or palliative settings. In this context, Voong et al. (2019) investigated the relationship between RT and ICI-Pneumonitis in patients with advanced NSCLC. Analysis of RT data included intent (curative vs. palliative), dose (Gy), number of fractions, treatment site (chest vs. non-chest), RT technique, and dosimetric parameters (mean lung dose and percentage lung volume receiving 20Gy). Notably, no specific RT treatment parameter was identified that increased the risk for the development of ICI-Pneumonitis. However, on subset analysis, patients who had received curative doses of chest radiation followed by ICI-based therapy were more likely to develop ICI-Pneumonitis (Voong et al., 2019).

The PACIFIC trial (Antonia et al., 2018) investigated the efficacy of definitive chemo-radiation followed by 1 year of consolidation with durvalumab (PD-L1 inhibitor) versus placebo on progression-free survival (PFS) and overall survival (OS) in patients with locally advanced, unresectable NSCLC. Sub-analysis of the prevalence of pneumonitis revealed that while there was a higher rate of all-grade pneumonitis in the durvalumab cohort, no difference was found in the rates of ≥ grade 3 pneumonitis (3.4 vs. 2.6%, no p-value).

In addition, the phase 1 KEYNOTE-001 study investigated the efficacy and safety of pembrolizumab (PD-1 inhibitor) in patients with advanced NSCLC (Garon et al., 2019). A secondary analysis of the data from this study, undertaken by Shaverdian et al. (2017), revealed that the duration of OS was longer (10.7 vs. 5.3 months) in patients who had previously received RT. Of the 24 patients who had previously received thoracic RT, 63% developed pulmonary toxicity versus 40% in the cohort who had not. Notably, there was no difference between the two groups with respect to the number of patients who developed ≥ grade 3 pulmonary toxicity (Shaverdian et al., 2017).

In patients receiving combined modality treatment, it becomes challenging for clinicians to differentiate whether pneumonitis is due to immunotherapy, RT, or a combination of the two. A study by Suresh^b^ et al. designed to investigate this issue established that the median time to ICI-Pneumonitis development was 82 days after the initiation of ICI, which is similar to the onset of radiation pneumonitis (Suresh et al., 2018b). In addition, the overlapping symptoms and radiographic features of radiation pneumonitis and ICI-Pneumonitis can make it difficult for clinicians to distinguish which type of pneumonitis a patient has developed. In this context, it is noteworthy that radiation pneumonitis usually occurs in, or in close proximity to, the irradiated field, while ICI-Pneumonitis most commonly develops at the edge of the radiation field or in a non-irradiated region (Voong et al., 2019). It is crucial to consider the effects of prior radiation therapy when evaluating a patient for suspected ICI-Pneumonitis.

### Diagnosis of Immune Checkpoint Inhibitor-Pneumonitis

Establishment of temporal eligibility (symptom development following initiation of the drug) and knowledge of typical symptoms and the usual latency period following drug exposure help to raise suspicion of ICI-Pneumonitis. Multi-disciplinary collaborations involving physicians with expertise in pulmonary medicine and infectious diseases and radiologists may expedite diagnosis and facilitate uniform management strategies. Diagnostic confirmation requires the exclusion of competing disease entities such as disease progression, infection, or thromboembolism. Thoracic computed tomography permits the identification of radiographic patterns of lung abnormalities and is the preferred imaging strategy. Analysis of blood, sputum, urine, nasal swabs and bronchoscopically-obtained lavage fluid may help to exclude infection. Evaluation for infection may be tailored to the index of suspicion for infection and the grade of suspected pneumonitis at presentation (Suresh et al., 2018a). Multiple society guidelines now advocate bronchoscopy with bronchoalveolar lavage and a complete infectious panel to evaluate patients with pneumonitis grades 2 or higher prior to initiation of immunosuppressive treatment. Bronchoalveolar lavage (BAL) fluid specimens typically demonstrate a lymphocytic pleocytosis (Suresh et al., 2019a). Lung biopsies are not a usual requirement for the diagnosis of ICI-Pneumonitis, but may assist in excluding other diagnoses, such as tumor progression. Histologic descriptions of ICI-Pneumonitis range from NSIP, OP, granulomatous inflammation, and cellular interstitial pneumonitis. Less commonly, acute fibrinous OP and diffuse alveolar damage are reported (Naidoo et al., 2017; Wang et al., 2021). Associated inflammatory infiltrates are typically lymphocyte-predominant. Poorly formed granulomas and increased numbers of eosinophils may also be seen (Naidoo et al., 2017; Hattori et al., 2019; Jodai et al., 2019). Increased levels of interleukin-6 (IL-6), tumor necrosis factor-α (TNF-α), interleukin-17 (IL-17), and to a lesser extent, interleukin-8 (IL-8) and interleukin-10 (IL-10) represent nonspecific signals of inflammation, but cannot distinguish inflammation in the setting of infection from a drug-related inflammatory response (Larsen et al., 2019; Wang et al., 2021). Differential expression of IL-17 in BAL IFNγ^+^ IL-17^-^ CD8^+^ T and CXCR3^+^ CCR6^+^ Th17/Th1 cells was noted in BAL fluid of patients with ICI-Pneumonitis compared to patients with pneumonia; however, larger studies are needed to confirm these findings (Kim et al., 2021). If available, changes in pulmonary function tests (PFTs) and 6-min walk tests (6 MWT) from baseline may be helpful in detecting lung function changes in ICI-treated patients. In addition, these studies may assist in risk stratification of patients with pre-existing lung disease and determine the severity of pneumonitis, while guiding response to specific ICI-Pneumonitis therapy. These tests have been incorporated into multiple national guidelines as simple, inexpensive, and non-invasive monitoring tools that can be performed at baseline before initiating ICI-based therapies (Brahmer et al., 2021). However, as demonstrated with other forms of drug-induced pneumonitis, the sensitivity of PFTs and 6 MWT in the early detection of ICI-Pneumonitis has not been established.

## Management of Immune Checkpoint Inhibitor-Pneumonitis

Management principles have not been validated in any prospective clinical trials and are primarily based on observational reports, clinical experience, and consensus opinion. Guidelines to assist in the diagnosis and management of ICI-Pneumonitis have been issued by several national and international societies, including the National Comprehensive Cancer Network (NCCN), American Society of Clinical Oncology (ASCO), American Thoracic Society (ATS), Society for Immunotherapy of Cancer (SITC), and European Respiratory Society (ERS) and the European Society for Medical Oncology (ESMO). These treatment strategies largely depend on the clinical severity of toxicity following the CTCAE classification scheme (Table 4) (Haanen et al., 2017; Brahmer et al., 2018; Sears et al., 2019; Brahmer et al., 2021).

**TABLE 4:** ICI-P: Diagnosis and treatment guidelines.

Grade of pneumonitis	Grade 1	Grade 2	Grade 3	Grade 4
Definition	Asymptomatic chest imaging findings	Mild symptoms that limit ADLs	Severe debilitating symptoms that limit self-care	Life-threatening symptoms
Intervention indicated?	No	Yes	Yes	Yes
FOB/BAL	FOB typically not indicated	Yes	Yes, if stable	Yes, when stable
PFTs, 6MWT	Consider at baseline and in 3–4 weeks	Yes	Yes, at baseline if stable and following discharge	Yes, at baseline if stable and following discharge
Treatment	• May continue ICI therapy uninterrupted with close monitoring	• Hold drug	• Discontinue drug	• Discontinue drug
		• Typically treated as outpatient	• Hospitalize	• Hospitalize
		• Start steroids (prednisone 1–2 mg/kg/day PO or methylprednisolone 1 mg/kg/day IV)	• Supplemental oxygen	• Supplemental oxygen, NIPPV
		• Consider antibiotics	• May require, NIPPV	• Typically requires ICU care, NIPPV, intubation
		—	• Start steroids (methylprednisolone 1 mg/kg/day IV)	• Start steroids (methylprednisolone 1 mg/kg/day IV)
		—	• Add antibiotics	• Add antibiotics
Follow-up	• Reassess after 3–4 weeks	• Close monitoring	• Close monitoring	• Close monitoring
	• If completely resolved or non-drug-related: continue treatment	• Symptoms improving: slowly taper steroids	• No symptom improvement or symptoms worsening after 48 h: add additional immunosuppressive therapy (infliximab, cytoxan, mycophenolate, tocilizumab)	• No symptom improvement or symptoms worsening after 48 h: add additional immunosuppressive therapy (infliximab, cytoxan, mycophenolate, tocilizumab)
	• If worsening: hold ICI and treat as grade 2 or 3/4	• Symptoms worsening: treat as grade 3/4	—	—
Duration of treatment	N/A	6 weeks	6 weeks, minimum	6 weeks, minimum
Restart Therapy	Yes	May be considered if symptoms resolve to grade 1	No	No

ADL = activities of daily living; BAL = bronchoalveolar lavage; FOB = fiberoptic bronchoscopy; ICU = intensive care unit; IV = intravenous; PFT = pulmonary function test; PO = by mouth; RX = therapy; NIPPV = noninvasive positive pressure ventilation; 6MWT = Six Minute Walk Test.

No specific intervention is recommended for patients with grade 1 pneumonitis, and ICI therapy may continue uninterrupted. Patients should be carefully monitored with repeat chest CT and lung function studies in 3–4 weeks. If symptoms or radiologic changes develop that presage disease progression, patients should be treated for higher-stage pneumonitis. For patients with grade 2 pneumonitis, withholding ICI therapy and initiation of oral prednisone, 1–2 mg/kg of ideal body weight/day, is recommended. These patients are typically monitored as outpatients with assessments within 48 h of initiation of steroid therapy and 1–2 times per week thereafter. Steroids should be continued at the initial dose until symptoms have returned to grade 1 (asymptomatic CT abnormalities) or to baseline, at which time steroids are slowly tapered over a 6-week period. Resumption of ICI therapy following successful treatment of grade 2 pneumonitis may be considered when there is resolution (or regression to baseline) of clinical and radiographic findings, steroid dose is less than 10 mg/day, and the patient is not receiving any other immunosuppressive therapy for ICI-Pneumonitis. Grades 3 and 4 pneumonitis are characterized by severe symptoms requiring hospitalization and prompt discontinuation of ICI therapy. With the exception of the European Society for Medical Oncology, which advocates 2–4 mg/kg of daily systemic steroids for patients in this group, most thoracic societies recommend 1–2 mg/kg/day of intravenous methylprednisolone or its equivalent for patients with grade 3–4 pneumonitis (Brahmer et al., 2021). The efficacy of the higher steroid dose has not been validated (Haanen et al., 2017; Brahmer et al., 2018). Intravenous methylprednisolone may be transitioned to oral prednisone once clinical improvement is established (typically after 48–72 h) with plans for a slow steroid taper over at least 6 weeks. Rebound pneumonitis following rapid steroid taper may be more severe than the initial presentation. ICI therapy should be permanently discontinued following grade 4 pneumonitis. The safety of ICI rechallenge after resolution of grade 3 pneumonitis has been debated, but is not recommended in most cases (Haanen et al., 2017; Brahmer et al., 2018). Evidence-based guidelines for ICI resumption following ICI-Pneumonitis have not been established, and decisions to re-treat should be systematically discussed in the context of a multidisciplinary team, calibrated against the risk: benefit ratio for each patient. ICI therapy should be permanently discontinued following grade 4 pneumonitis.

If an infectious cause remains a possibility following the diagnostic workup for ICI-Pneumonitis, empiric broad-spectrum antimicrobial therapy, given in conjunction with immunosuppressive therapy, is advocated (Haanen et al., 2017; Brahmer et al., 2018). Emerging data suggests that the gut microbiome plays a substantial role in the efficacy of ICI-based therapy (Piccinno et al., 2014; Derosa et al., 2018; Zitvogel et al., 2018; Elkrief et al., 2019; Derosa and Zitvogel, 2020). Antibiotics are known to alter the gut microbiome (Zimmermann and Curtis, 2019). Thus, the role of empiric antibiotics in this scenario will need to be calibrated against growing concerns regarding the potential adverse effects of antibiotic therapy on ICI efficacy.

Clinical improvement is seen in most patients with low-grade pneumonitis, although imaging abnormalities may persist well beyond clinical recovery (Johnson et al., 2019; Fukihara et al., 2019). Reported mortality rates associated with grade 3–4 ICI-Pneumonitis are high (22–33%), highlighting the need for early detection and optimization of treatment (Wang et al., 2018; Tone et al., 2019). Opportunistic infections triggered by prolonged high-dose steroid therapy are thought to be an important cause of ICI-Pneumonitis-related major morbidity and potentially mortal (Wang et al., 2021)). Patients that show no signs of clinical or radiographic improvement after 48 h of steroid therapy are deemed to be steroid-refractory. Guidelines in these patients are based on expert consensus and include the use of infliximab, mycophenolate mofetil, cyclophosphamide, intravenous immunoglobulin, or tocilizumab (Haanen et al., 2017; Brahmer et al., 2018; Stroud et al., 2019; Brahmer et al., 2021). Both infliximab and cyclophosphamide have been approved by the US FDA for patients with digestive toxicities associated with ipilimumab, while possible benefits of IL-17 blockade in the management of immune-mediated gastrointestinal and skin toxicities were also suggested in a recent report (Esfahani and Miller, 2017). High levels of IL-17 have also been demonstrated in BAL fluid and peripheral blood among patients with ICI-Pneumonitis, indicative of the therapeutic potential of monoclonal antibody targeting of this cytokine or its receptor in this irAE (Petri et al., 2019; Stroud et al., 2019; Kim et al., 2021; Tyan et al., 2021). However, robust data regarding the use of any of these agents in ICI-Pneumonitis is currently not available. It is, nevertheless noteworthy that the IL-6 receptor inhibitor, tocilizumab, has shown promise as a therapeutic option in steroid-refractory toxicities associated with immune checkpoint blockade, including ICI-Pneumonitis. However, prospective clinical trials are needed to fully elucidate the efficacy of tocilizumab therapy in this setting (Stroud et al., 2019).

### Acute Immune Checkpoint Inhibitor-Mediated Pulmonary Emergencies

In conjunction with corticosteroid therapy, it is essential that the patient receives appropriately targeted oxygen therapy and chest physiotherapy to support good sputum drainage. Judicious empirical antimicrobial therapy in accordance with local guidelines should be commenced while infection screens are being processed. Early decisions regarding escalations of care, including whether intubation and ventilation should be undertaken.

Other rare immune-mediated respiratory emergencies include myositis of the diaphragm, which presents with rapidly progressive neuromuscular hypoventilation (Haddox et al., 2017; John et al., 2017). In accordance with the management of other life-threatening, immune-mediated toxicities, these patients require early recognition with the initiation of high-dose corticosteroids, early immunosuppression, and critical care support.

Pulmonary embolism (PE) is more common in melanoma patients treated with ICIs (Sussman et al., 2021). Furthermore, it appears to be more prevalent in patients treated with combination checkpoint inhibition in comparison with single-agent therapy. A high clinical suspicion for PE is thus required in dyspnoeic patients with no other apparent cause, and therapeutic anticoagulation should be commenced after confirmation of diagnosis.

Rare pulmonary complications associated with nivolumab include acute pulmonary hypertension and an increased pulmonary artery diameter (Fournel et al., 2020).

COVID-19 may mimic presentations of ICI- Pneumonitis and is a recent important addition to the differential diagnosis in patients with acute respiratory symptoms treated with ICI therapy (Cooksley et al., 2021).

### COVID-19 and Immune Checkpoint Inhibitor-Pneumonitis

The COVID-19 pandemic caused by severe acute respiratory syndrome coronavirus 2 (SARs-COV-2) has disproportionately affected patients with cancer (Bakouny et al., 2020; Garassino et al., 2020). Weakened T cell activity caused by exhaustion of T lymphocytes is a distinguishing feature of cancer and chronic infections. Characteristic findings of exhausted T-lymphocytes include overexpression of PD-1 and CTLA-4 immune checkpoint receptors and loss of IL-2 production, impaired cytotoxicity, diminished proliferation, and altered production of proinflammatory cytokines. T cell exhaustion is a hallmark of COVID-19 infection (Dyck and Mills, 2017; Hashimoto et al., 2018; Wykes and Lewin, 2018). ICIs can abrogate T cell exhaustion and depletion by blocking CTLA-4 and PD-1 inhibitory signaling. These observations suggest that cancer patients with concomitant infection might derive benefit from ICI therapy.

On the other hand, probable upregulation of hitherto suppressed positive immune checkpoint molecules in effector T cells and resultant generalized inflammatory cytokine secretion may exacerbate end-organ damage associated with cytokine release syndrome, leading to more severe COVID-19 symptoms. These observations have raised concerns regarding the safety of ICI therapy for cancer patients in the setting of COVID-19. The TERAVOLT study, a multinational investigation of 200 patients with thoracic cancer and COVID-19, reported that the mortality associated with COVID-19 infection in patients with active thoracic cancers was 33%. The type of systemic therapy (tyrosine kinase inhibitor, conventional chemotherapy, or ICI therapy) did not impact survival, suggesting that withholding or discontinuing ICI therapy for cancer patients with concomitant COVID-19 might not be warranted (Garassino et al., 2020). Several other smaller studies corroborate these findings (Kattan et al., 2020; Luo et al., 2020; Quaglino et al., 2020). These results must be considered with caution, and further prospective investigations are needed.

Cytokine release syndrome (CRS) is very rarely observed following ICI therapy and appears clinically indistinguishable from a cytokine storm, a key feature of advanced COVID-19. However, overlapping clinical features of ICI- Pneumonitis, which is much more common, and COVID-19 pneumonitis create a more frequent conundrum, rendering accurate diagnosis and appropriate management difficult. Systemic glucocorticoids are the mainstay of treatment for grade 2 or higher ICI-Pneumonitis and during the early stages of COVID-19 pneumonia with ARDS. Cytokine excess, in particular IL-2, IL-4, IL-6, TNF-α, and IFN-Ɣ are known drivers of the overactive inflammatory response and associated COVID-19 severity (Liu et al., 2020; Du et al., 2021). The TNF-α inhibitor, infliximab, and the IL-6 inhibitor, tocilizumab, are used as second-line therapies in patients with steroid-refractory irAEs, including ICI-Pneumonitis. Their role in the management of COVID-19 is under intense investigation, with early evidence from the REMAP-CAP trial showing therapeutic promise (Gordon et al., 2021).

## Other Manifestations of Immune Checkpoint Inhibitor-Related Lung Injury

### Sarcoid-like Reactions

**FIGURE 3:**
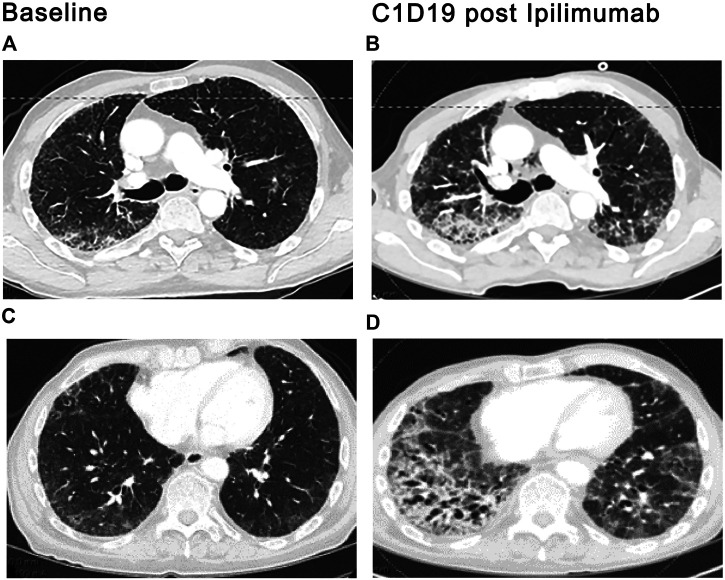
ICI-related sarcoid-like reaction. Diffuse lymphadenopathy in paratracheal, bi-hilar, and mediastinal locations (arrows), **(A,C)**, developed after the third cycle of ipilimumab plus nivolumab therapy for bladder cancer. Diffuse interstitial and nodular infiltrates along bronchovascular bundles (arrows), **(B,D)** were also seen. Transbronchial biopsies and biopsies of the lymph nodes by endobronchial ultrasound (EBUS) demonstrated noncaseating granulomas. The diagnosis of ICI induced sarcoid-like reaction was confirmed once competing diagnoses were excluded.

Both CTLA-4 and PD-1/PD-L1 inhibitors have been added to a growing list of agents implicated in developing granulomatous syndromes indistinguishable from sarcoidosis (Vogel et al., 2012; Kim et al., 2016). Although mechanistic underpinnings of ICI-associated sarcoid-like reactions have not been fully elucidated, amplification of Th17 by CTLA-4 and PD-1 axis blockade is thought to play an integral role (Vogel et al., 2012; Huang et al., 2013; Braun et al., 2014; Broos et al., 2015). Noncaseating granulomas, the cardinal feature of sarcoid-like reactions, are indistinguishable from confirmed sarcoidosis. Sarcoid-like reactions most commonly involve the lungs, lymph nodes, and skin. Other extrathoracic manifestations involving the parotids, spleen, bone, eyes, and brain have also been reported. Patients typically present at 12 weeks (range 3 weeks to 2 years) following drug administration with asymptomatic mediastinal and/or hilar lymphadenopathy and upper lobe predominant pulmonary nodules in a perilymphatic distribution on CT scan (Figure 3) (Kim et al., 2016; Rambhia et al., 2019). Dry cough and dyspnea are occasionally reported. No apparent ICI dose threshold that triggers sarcoid-like reactions has been identified. ICI-related sarcoid-like reactions show fluorodeoxyglucose uptake (FDG) in involved tissues on positron-emission tomography (PET) scans that are indistinguishable from confirmed sarcoidosis and sarcoid-like reactions from other causes of lymphadenopathy, including malignancy. Hypercalcemia and elevations in serum angiotensin-converting enzyme levels have also been observed (Kim et al., 2016). Pulmonary function studies are typically normal. BAL fluid commonly demonstrates a CD4-rich lymphocytic alveolitis. The diagnosis requires examination of tissue samples obtained by bronchial biopsy, transbronchial lung biopsy, or biopsies of the mediastinal lymph nodes using endobronchial ultrasound (Luke et al., 2015). Findings of focal infiltration by noncaseating epithelioid and giant cell granulomas establish the diagnosis once other causes have been excluded. In most patients, resolution of signs and symptoms following drug-interruption occurs with or without the initiation of steroids. Spontaneous resolution in early disease despite continuation of ICIs has also been reported. Recurrence of sarcoid-like reactions following resumption of ICI therapy may occur. The indication, dose, and duration, and optimal tapering schedule of steroid therapy in this context has not been defined (Berthod et al., 2012; Murphy et al., 2014; Danlos et al., 2016).

### Radiation Recall

**FIGURE 4:**
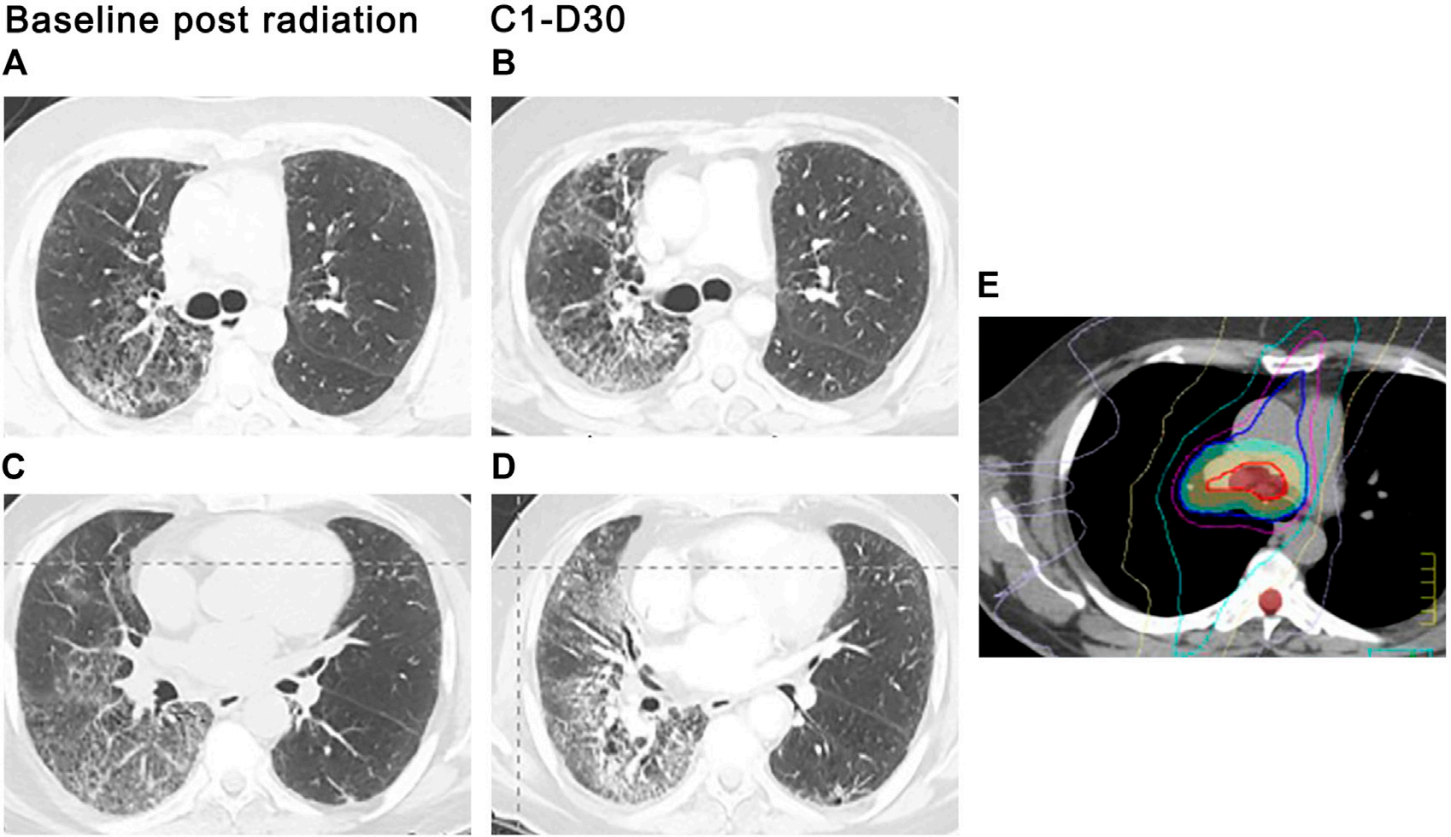
ICI-related radiation recall. A 56-year-old man with metastatic adenocarcinoma of the lung presented with severe shortness of breath and dry cough 3.5 weeks after the first cycle of pembrolizumab for metastatic adenocarcinoma. Radiation therapy to the right chest was completed 13 months prior. Stable subpleural reticular opacities associated with traction bronchiectasis are seen on the baseline CT and consistent with sequelae of prior radiotherapy **(A,C)**. Admitting CT scan demonstrated new ground glass opacities in the right upper and lower lobes **(B,D)** that conform with the prior radiation portal **(E)**. Centrilobular emphysema is again seen. Findings suggest radiation recall precipitated by pembrolizumab therapy.

Specific drugs may trigger radiation injury within a previously irradiated field. This phenomenon, known as radiation recall pneumonitis (RRP), may be provoked by a variety of agents, including the taxanes, anthracyclines, gemcitabine, erlotinib, etoposide, oxaliplatin, and vinorelbine (Burris and Hurtig, 2010; Ding et al., 2011). More recently, RRP has been reported following anti-PD1 therapy (Gupta and Lee, 2017; Shibaki et al., 2017). In one study, the incidence of PD-1-triggered RRP among patients with NSCLC was 4.2% but rose to nearly 31% among patients with a prior history of PD-1 related pneumonitis (Gupta and Lee, 2017). The development of new ground-glass and consolidated opacities within the irradiated field signals RRP development, which typically appears 3 months to 2 years after completion of radiation therapy and one to 2 months after exposure to the triggering chemotherapeutic agent (Gupta and Lee, 2017; Shibaki et al., 2017). The pulmonary infiltrates may be asymptomatic or associated with dry cough, dyspnea, chest pain, and low-grade fevers (Figure 4). Signs and symptoms typically respond to systemic corticosteroids. We use 0.5–1 mg/kg of methylprednisolone or equivalent with a 4–6-weeks taper, although the optimal dose and duration of steroid therapy have not been established in randomized trials.

### Pseudoprogression and Hyperprogression

Pseudoprogression represents an unconventional clinical response to ICI therapy in which there is an initial increased size of tumor lesions or the appearance of new lesions on CT, with subsequent reduction in tumor burden. This clinical response typically occurs during the first cycles of therapy and has been observed in 2.8–15.8% of all ICI-treated patients across tumor types. Pseudoprogression may be erroneously classified as progressive disease according to the size-based WHO or RECIST criteria, leading to premature discontinuation of the drug. Distinguishing pseudoprogression from true tumor progression based on radiological evidence alone is challenging, particularly early in the course of ICI therapy. Ideally, pathologic findings of peritumoral necrosis or inflammatory cell infiltrates are needed for confirmation; however, tissue biopsies may not be pragmatic in some patients. Thus, the diagnosis of pseudoprogression is based on CT findings of tumor enlargement following the initial cycle of ICI blockage and subsequent shrinkage in tumor burden on subsequent CT, performed at least 4 weeks later. If tumor shrinkage is noted on the subsequent CT, ICI therapy may be safely continued. Shrinkage of tumor elsewhere and the absence of clinical deterioration in the patient’s condition supports the diagnosis of pseudoprogression.

Unlike pseudoprogression, in which there is a radiographically apparent increase in tumor burden after ICI therapy followed by tumor regression, hyperprogression, represents true tumor progression in which there is a very rapid and sustained progression of tumor following the initiation of immunotherapy. This ICI-related rapid surge in tumor burden has primarily been reported following PD-1/PD-L1 therapies for lung cancer (Kato et al., 2000; Chubachi et al., 2016; Kurzrock and Kato, 2018; Champiat et al., 2019).

### Airway Disease

Severe isolated airway diseases, including bronchitis and bronchiolitis, have been attributed to ICI therapy in individual case reports. Response to bronchodilator therapy and inhaled steroids has been variable (Mitropoulou et al., 2020). Pleural effusion has also been rarely described following PD-1 and PD-L1 therapies (Kolla and Patel, 2016; Ikematsu et al., 2019). Effusions are lymphocyte-predominant and may spontaneously remit despite continuation of ICI therapy.

## Conclusion

In the wake of the rapidly expanding field of cancer immunotherapeutics, knowledge regarding immune-related adverse events and toxicity profiles of various organ systems is slowly starting to emerge. Lung toxicity associated with this class of agents is no exception. Over the past decade, since the first ICI was FDA-approved in the United States, it has become apparent that the 2–5% incidence of ICI-related lung injury reported in early clinical trials grossly underestimates the real-world experience of 12–19%. ICI grading systems endorsed by national organizations have helped characterize ICI-Pneumonitis better and establish diagnostic evaluation and management standards. Much of the data examining ICI-Pneumonitis has been gleaned primarily from retrospective reports, but these have significantly increased our understanding of the clinical manifestations and temporal onset of disease. Recognition of stereotyped patterns of ICI-related lung injury on imaging studies has facilitated earlier diagnosis. Identification of vulnerable populations, toxic drug-drug combinations, and adverse drug interactions with other treatment modalities has been critical for risk stratification and optimizing treatment outcomes. However, key issues and fundamental research questions remain.

First and foremost is the lack of a consensus definition of pneumonitis and validated diagnostic criteria. Without these factors, ICI-Pneumonitis is subject to widely varying definitions and inaccurate reporting. Further clarification of at-risk populations and ICI treatment combinations that confer a higher risk for lung injury is also needed. Finally, identifying biomarkers and improvements in diagnostic strategies may help refine the diagnosis and facilitate the timely initiation of appropriate treatment.
